# Internet-Delivered Self-help for Adults With ADHD (MyADHD): Usability Study

**DOI:** 10.2196/37137

**Published:** 2022-10-21

**Authors:** Robin Maria Francisca Kenter, Adrian Schønning, Yavuz Inal

**Affiliations:** 1 Department of Clinical Psychology Faculty of Psychology University of Bergen Bergen Norway; 2 Section for Research-Driven Innovation Division of Psychiatry Haukeland University Hospital Bergen Norway; 3 Department of Design Faculty of Architecture and Design Norwegian University of Science and Technology Gjøvik Norway

**Keywords:** usability testing, user evaluation, attention-deficit/hyperactivity disorder, self-guided intervention, internet-delivered, self-help, adults with attention-deficit/hyperactivity disorder, intervention, usability study, care needs, usability

## Abstract

**Background:**

Although effective pharmacological treatment exists, many adults with attention-deficit/hyperactivity disorder (ADHD) prefer a nonpharmacological option for managing their symptoms. Internet-delivered self-help interventions have the potential to address this unmet supportive care need reported by adults with ADHD, at relatively low costs. However, if the intervention does not offer optimal functions, content, and layout, it could decrease adherence and engagement and potentially compromise the effectiveness of such interventions. Thus, there is a need for examining the usability and factors that enhance and impair the usability of internet-delivered self-help interventions.

**Objective:**

This study evaluates the usability of an internet-delivered self-help intervention for adults with ADHD (MyADHD). The main goals were to (1) collect qualitative and quantitative data on usability and (2) identify usability problems.

**Methods:**

Individual think-aloud interviews and staged usability testing (N=5) were conducted to evaluate the usability of the MyADHD intervention in terms of function, content, and design. MyADHD end users provided iterative feedback to maximize engagement and usability. They performed tasks involved in operating the intervention and provided “think-aloud” commentary and postsession usability ratings. The interviews were recorded, transcribed verbatim, and analyzed.

**Results:**

Participants were satisfied with the overall usability of the program. The average perceived usability score out of 100 was 70 for the first round of testing and improved to 77.5 after applying modifications, with a mean score of 75.5 (SD 5.9) for all rounds of usability testing. The analysis of the interviews revealed 3 central themes: functionality, content, and layout.

**Conclusions:**

Optimizing the usability of internet-delivered self-guided interventions is a critical step in the design and development process. The usability testing in this study provided valuable information from users’ perspectives on the content and platform of the intervention. Analysis revealed the need for intervention enhancement with regard to design, functionality, and content from the perspective of potential end users. Overall, participants saw value in the MyADHD intervention and were confident that they could use it for the self-management of symptoms and expressed the desire to use the entire intervention when it becomes available. Through this development process, we produced an intervention that is likely to be used successfully and is ready for deployment in a randomized controlled trial.

**Trial Registration:**

ClinicalTrials.gov NCT04511169; https://clinicaltrials.gov/ct2/show/NCT04511169

## Introduction

### Background

Digital technology for the treatment and management of mental health issues has become increasingly available in recent years [1]. For common mental health disorders like depression and anxiety, internet-delivered interventions are part of official treatment guidelines [2]. Internet-delivered interventions either can be delivered with some form of guidance or can be entirely self-guided [3]. Effective internet-delivered self-guided interventions can dramatically increase treatment accessibility at relatively low costs, as they can be distributed over the internet directly to the user’s computer, without the need for therapist guidance [4]. However, in self-guided internet-delivered interventions, a commonly described issue is low adherence and engagement [5].

### Internet-Delivered Interventions for Adults With ADHD

For people with attention-deficit/hyperactivity disorder (ADHD), few evidence-based internet-delivered interventions for adults exist today. ADHD in adults is characterized by pervasive symptoms of inattention, hyperactivity/impulsiveness, or both that persist across different situations [6]. With an estimated prevalence of 2%-3%, ADHD can be counted as a common mental disorder in adulthood [7-9]. Although effective pharmacological treatment exists [10], many adults with ADHD prefer a nonpharmacological option for managing their symptoms [11]. In this case, self-guided psychological interventions that can be delivered over the internet might have the potential to meet the treatment preferences of adults with ADHD. These types of interventions can help manage symptoms of ADHD by providing general information, tailored advice, support, and skill training via technology (eg, smartphones, tablets, or websites). The few studies that examine internet-delivered psychological treatments for adults with ADHD show good clinical outcomes [12-14]. Self-guided internet-delivered interventions for adults with ADHD have the potential to offer evidence-based interventions to many individuals, with 24/7 access to information, in the comfort of their own homes, where they can read and reread materials.

However, self-guided interventions are also associated with higher dropout rates than guided interventions. Since adherence is related to outcome, it is important to focus on all factors that could improve adherence to self-guided internet-delivered interventions. This starts with designing good systems that address the needs of the end users and have good usability. The primary reasons for low engagement with internet-delivered interventions has been a lack of user-centered design and poor usability [15]. Interventions with poor usability may make it difficult to interact with the intervention, which could lower the engagement, which then can lead to suboptimal clinical outcomes or rejection of the intervention by the user. This might be especially a problem for adults with ADHD, where the cardinal symptoms often are inattention and impulsiveness. It is, therefore, important to test the usability of a new internet-delivered intervention for ADHD during the development process, so that usability flaws are known at an early stage and can be fixed before the intervention goes to full trial.

### Usability

Usability is defined by the International Organization for Standardization [16] as “the extent to which a product can be used by specified users to achieve specified goals with effectiveness, efficiency and satisfaction in a specified context of use.” It also refers to the quality of a user's experience when interacting with systems, including websites and mobile apps. The usability of an internet-delivered intervention has a direct influence on user satisfaction. When an internet-delivered intervention is not well designed, it can prevent users from effectively and efficiently using the program. Usability evaluation is a very valuable resource to understand how easy it is to use a developed system for new users. It can be summative (collecting evidence that the intervention is usable) or formative (informing the redesign and improvement of the intervention) [17].

This study describes the results from a formative usability evaluation of the MyADHD intervention [18] that gathered feedback from end users and improved the design as part of an iterative design process.

### Formative Usability Evaluation of MyADHD

In this study, we evaluated the usability of a first prototype of an internet-based intervention for adults with ADHD. The main goals were to (1) collect qualitative and quantitative data on usability and (2) identify usability problems. An iterative development process was used to promote the further development of the content, visual design, and interaction design. Once the first round of usability testing was completed, we evaluated, determined improvements, implemented the improvements, and retested the updated prototype. We included 5 rounds with usability testing with 5 potential end users, as 5 users are often enough to identify 80% of all usability problems [19]. The goal was to identify understandability, ease of learning the intervention’s platform, and appropriateness of the intervention. Through the testing, we were provided with feedback about what does and does not work in the intervention for end users and determined whether the features of the intervention were acceptable and feasible for users.

### Objectives

This study aims to investigate the usability of a self-guided internet-delivered intervention (MyADHD), which targets symptoms of ADHD among adults.

## Methods

### Procedures

The usability evaluation was performed in 2 parts: (1) the expert evaluation and (2) the user evaluation. This study reports on findings from the first stage of the formative research process: usability testing. The aim is to investigate the usability of a self-guided internet-delivered intervention in terms of function, content, and design.

#### Part 1: Expert Evaluation

The first and last author of the paper (RMFK and YI) conducted an expert evaluation prior to the user evaluation. Through this evaluation method, we detected usability problems with the interface early in the process [20]. The expert evaluation involved an evaluation on how well a design supported its target audience in achieving their specified goals with effectiveness, efficiency, and satisfaction. The usability expert (YI) conducted a review according to a set of generally accepted usability guidelines, as well as their personal knowledge of the design’s domain. The evaluation was based on Nielsen’s heuristics. Problems detected by the evaluators were addressed immediately, so they did not influence the second part: the user evaluation.

#### Part 2: User Evaluation

Five individuals diagnosed with ADHD were recruited via the Norwegian patient association (ADHD Norge) to participate in a 1-hour laboratory-based usability test. The inclusion criteria for the usability tests were (1) a self-reported ADHD diagnosis, (2) age >18 years, and (3) willing to participate and able to meet at the laboratory. In the laboratory, participants used a laptop and were asked to use the prototype of MyADHD intervention. The test entailed the completion of 10 goal-oriented tasks (see Textbox 1).

**Tasks.**

1. Go to the program (adhd.youwell.no) and log in
2. Start module 1, read the material, and say out loud what you think
3. Start module 2 and try one of the breathing exercises
4. Log your experience of the breathing exercise in the exercise log
5. Complete module 2
6. Go to the home page
7. Fill out the questionnaires of module 3
8. Go to my diary and fill out an entry
9. Go to my calendar page and see this week’s homework assignments
10. Try another breathing exercise and log your experience again

First, the participants provided demographic information and reported on their experience with personal computers and the use of internet. Next, they were asked to perform a series of tasks in the platform and intervention. Two facilitators were present in each usability session. One led the session and 1 observed. Participants were asked to “think aloud” (ie, provide continuous commentary) in accordance with Concurrent Think-aloud Method (CTA) while operating the system [21]. The goal with CTA was to encourage participants to keep a running stream of consciousness as they worked with the tasks. This helped to understand participants’ thoughts as they tried to work through the intervention and elicit real-time feedback and emotional responses to the intervention. This is a common approach to usability testing that enables evaluation of the ease with which a system is learned [22]. Participants provided feedback on the MyADHD intervention (ie, layout, color, text, readability, and videos), and the content and provided information on their preferences regarding the use of MyADHD. If the participant asked a question, the facilitator remained neutral and replied with “What do you think?” or “What would you do?”

During the CTA, the observer took notes and used an audio recorder to capture all that the participant did and said. At the end of the session, the System Usability Scale [23] was administered as an online survey in the platform. The information retrieved from the think-aloud interviews was used to further optimize the MyADHD intervention.

### Ethics Approval

This study was reviewed and approved by the Norwegian Regional Committee for Medical and Health Research Ethics, REC South East #203804. The participants provided written informed consent to participate in this study. As a reimbursement for their time, participants received gift vouchers worth NOK 200 (US $19).

### Questionnaires

#### The System Usability Scale

The System Usability Scale (SUS) [23] is a widely used source for the assessment of the perceived usability of an evaluated system [24]. The SUS has sufficient reliability (coefficient α .9). It contains 10 items with a response from 1 (strongly disagree) to 5 (strongly agree), with 5 positive statements and 5 negative statements. The items are scored on a 5-point Likert scale. The sum of the items leads to a general measure of perceived usability with the total score varying between 0 and 100. Based on different studies in the literature, an overall usability score of below 70 shows poor usability of the evaluated system [25,26]. Higher overall scores represent a high quality of usability. The scale allows item-based analysis by calculating the mean score and standard deviation of each item. Minor wording changes do not appear to affect SUS scores [25,27]. In our research, we tailored the scale by replacing the term “system” with “the intervention.”

Additionally, the participants were asked questions on sociodemographic and internet use characteristics.

### The MyADHD Intervention

MyADHD development was theory based and person based [28]. The planning phase consisted of qualitative focus groups, the development of guiding principles, and a literature review. The first prototype of the intervention was delivered via an online secure portal, which is accessible on laptops and personal computers. The intervention is a structured self-guided intervention with modified elements from cognitive behavioral therapy, dialectical behavioral therapy, and goal management training to target specific challenges experienced by adults with ADHD. Kenter et al [18] described the development of the content of the intervention. The intervention consists of 7 training modules. In this study, we used the first 3 modules of the intervention (see Multimedia Appendix 1).

The main goals of the intervention are to help participants obtain improved functioning in daily life activities and offer strategies that aim to reduce stress, reduce inattention, and improve quality of life. Each module includes psychoeducation alongside the text, audio, and video material instructing participants in the use of specific techniques. Further, modules include case vignettes and lived-experience videos that clarify the written content and help participants make connections between the material and their own experiences. Since all modules have the same structure and ingredients, we only used the first 3 modules in our test. Table 1 shows an overview of the intervention modules that were used in this study.

**Table 1:** Overview of the first 3 modules of MyADHD.

Module	Rationale and content	Exercises and videos
1. Start module	Goal setting and practical information about how to use the internet-delivered intervention	One goal setting exercise Describe how ADHD^a^ affects your life exercise One lived-experience video
2. Mindful awareness	Inattention is a core symptom of ADHD. In this module, participants are given information about different aspects of attention and concentration and how to cope with impairment. In this module, the participants start training on mindful awareness (“being here and now”) by focusing on their breathing (based on dialectical behavioral therapy).	Three different types of breathing exercises Two lived-experience videos
3. Inhibition training	Impulsivity and loss of impulse control are common among adults with ADHD. This module consists of exercises focusing on impulse control and goal-oriented/goal-directed behavior (stop, observe, proceed, and check; based on goal management training).	Two STOP exercises Two lived-experience videos

^a^ADHD: attention-deficit/hyperactivity disorder.

### Data Collection and Analysis

#### Tasks and Questionnaires

In each individual usability testing session, following informed consent, the participants were first asked to think aloud while using the program and doing the tasks (see Textbox 1). The procedure was pretested with an individual who was not involved in the study. A facilitator guided the participant through the usability sessions by presenting the tasks and reminding the participants to think aloud by questions like “Tell me what you’re thinking,” “what are you looking for?,” and “What do you think now?” The sessions were audiotaped and transcribed verbatim. We coded and analyzed the transcripts using NVIVO [29].

### Data Analyses

The usability tests were recorded and transcribed verbatim by the second author. Transcripts were analyzed thematically using an iterative coding procedure. The focus of the analysis was on the features of the intervention that needed to be redesigned or improved. The categories were identified using an iterative process of reading and rereading the transcripts. Usability issues were coded into categories.

## Results

### Participants

The participants had a mean age of 38.4 (SD 16.3) years. Three were men and 4 were highly educated (high school or higher). One participant reported difficulty using computers and the internet, while the others had reported good computer skills, see Table 2. All participants had ADHD diagnoses, and none of them had previously used an internet-delivered intervention before.

**Table 2:** Demographics.

				Value
**Demographics**				
	**Age (years)**			
		Mean (SD)	38.4 (16.3)	
		Range	25-62	
	**Gender, n**			
		Male	3	
		Female	2	
	**Educational level, n**			
		Middle	1	
		High	4	
**Internet and computer skills, n**				
	Self-reported good computer skills			4
	Self-reported good internet skills			4

### Usability Issues

The transcripts revealed 3 main categories of barriers that limited usability: (1) functionality, (2) content, and (3) layout.

#### Functionality

This category referred to the need for the intervention to be easy to use, navigate, and have a logical flow. Analysis showed that usability was limited when navigation was difficult. The participants experienced problems related to, for example, unclear navigation (“I can’t move back to the homepage,” “where do I go to see my past entries?”), unclear functionality (“Does the calendar synchronize with my own phone?”), and it was unclear whether filled-out questionnaires were saved and submitted (“Did I save this now? I am not sure, can I go back? How do I check this?”). Furthermore, textboxes were too small to type in larger amounts of text.

In preparation of new rounds of usability testing, back and forward buttons were added, more explanation was offered with the different elements such as calendar, automatic feedback that answers on questionnaires were sent in, and textboxes were made larger. At the end of usability testing, the participants found the calendar still confusing to use. Due to this, the calendar was removed from the intervention, and the participants were encouraged to use their own private calendar, for example, their mobile phone calendar or paper calendar. After this adjustment, think-aloud comments to the functionality were more neutral (“OK, now I click here to go to the next page”), positive (“Nice! I did not remember what I was supposed to do, but I can go back to read the previous page”), or focused on the specific intervention content.

#### Content

Overall, the participants had a general positive impression of the content in the program. The program felt “positive,” “light,” and “useful.” The exercises were perceived as helpful and useful (“This really speaks to me,” “this makes perfect sense”). Usability testing revealed that several participants (n=4) had difficulty understanding the text when it was too lengthy, with large blocks of text, making the information difficult to process, which limited the usability.

A point of improvement involved the wording in the content, whereby language was perceived as too negative (“I do not recognize myself in having problems, I might have challenges, not problems”) and too complicated (“I have to read the most sentences twice”). There were also inconsistencies in wording that made it difficult to use the intervention (“first you call it dashboard, and now I have to go back to the homepage,” “it is called a program or an intervention? It seems now that these are two different things”).

This resulted in changes in the wording; more positive wording was used, which resulted in more positive comments about the wording (“It is nice with empathy about that it can be difficult to be part of this ADHD intervention”). Wording was checked for inconsistencies, spelling errors were removed, and text was shortened or chunked up in smaller sections so that it became easier to read. We also added a short summary at the end of each module. All participants liked the videos, exercises, and psychoeducational text and viewed them as helpful.

#### Layout

All participants commented that they saw value in the images, visual aids, and videos. They liked the layout and described it as “calm,” “beautiful, yeah it is esthetic,” and “friendly” with “nice colors and pretty images.” Regarding the questionnaires, the participants found it hard to fill out the questionnaires because of the large amount of text and answering options.

### Satisfaction With the Intervention

All participants felt confident they would be able to use the platform (n=3 strongly agreed, n=2 agreed). The participants were satisfied with how easy it was to use and viewed the intervention as helpful. All participants were able to perform the tasks and learn to use the intervention and the platform on their own.

### Perceived Usability Outcomes

The results for perceived usability (as measured by the SUS) are presented in Table 3. The analysis of the data identified that the intervention was rated with an average score of 75.5 (SD 5.9). Of the 5 participants, 1 rated the MyADHD intervention as “excellent,” while 4 others rated it as “good.”

**Table 3:** Result of the System Usability Scale.

Items	P1^a^	P2	P3	P4	P5	Mean
1. I think that I would like to use this intervention	4	3	3	4	3	3.4
2. I found the intervention unnecessarily complex	2	2	2	1	1	1.6
3. I found the intervention easy to use	4	4	4	5	5	4.4
4. I think I would need support from a technical person to use this intervention	1	2	1	1	1	1.2
5. I found the various functions in this intervention were well integrated	3	4	4	3	3	3.4
6. I thought there was too much inconsistency in this intervention	3	2	2	3	3	2.6
7. I would imagine that most people would learn to use this intervention quickly	3	3	4	5	4	3.8
8. I found the intervention very cumbersome to use	2	2	2	1	2	1.8
9. I felt very confident using the intervention	3	5	3	4	5	4
10. I needed to learn a lot of things before I could start using this intervention	1	2	2	1	2	1.6
SUS^b,c^ score (0-100 scale)	70	72.5	72.5	85	77.5	75.5

^a^P1-5: participant 1-5.

^b^SUS: System Usability Scale.

^c^Total score reversed to a 0-100 scale.

## Discussion

### Principal Results

Usability testing demonstrated that all individuals could perform the desired tasks, and that they learned to use the intervention quickly. Results from the SUS revealed that the program obtained high scores, indicating that the intervention was considered very useful. Staged iterative usability testing was essential for discovering intervention enhancement needs (eg, more visual aids, more buttons) and for resolving design, functionalities (eg, more feedback on actions and explanation to functionalities), and content inadequacy (eg, inconsistencies, difficult wording, lengthy text). Overall, the participants saw value in the MyADHD intervention and were confident they could use it for self-management of symptoms and expressed the desire to use the entire intervention when it becomes available.

Optimizing usability early in the process is a critical step in the development process of self-guided internet-delivered interventions [28], especially with self-guided interventions, where uptake and adherence are often low [4]. While there is a large number of studies examining the feasibility and efficacy of internet-delivered interventions for common mental health disorders like depression and anxiety [1], there are only a few studies that examine internet-delivered interventions for adults with ADHD [12,13,30]. These studies reported low adherence to the interventions. This is problematic as adherence is related to outcome [31,32]. Additionally, low usability could have an impact on the effectiveness of such interventions [33]. A study on usability is therefore important and relevant, as it may help to ensure that the interventions are well designed and therefore increase the interest and number of people who can benefit from it. End users have high expectations of digital products, and because of difficulties with inattention, adults with ADHD specifically might drop out if the intervention does not have optimal usability. We did not find other studies examining the usability of internet-delivered interventions for adults with ADHD.

The overall SUS scores found in this study are comparable to other usability studies of internet-delivered interventions. A study of transdiagnostic internet-delivered treatment [34] found a mean SUS score of 81.89; a study on blended care [35] found a mean SUS score of 76.3 for the internet-delivered treatment, while a study on web-based support for people with mild intellectual disabilities [36] found a low mean SUS score of 56.4 and 51.1.

### Limitations and Strengths

For usability tests, 5 users are often enough to identify 80% of all usability problems [19]. The optimal number of participants required is not clear. This depends on the problems raised by the participants and whether the participants give an adequate reflection of potential end users. Despite the small sample, participants with different profiles and backgrounds were included, and within this sample, theme saturation was achieved. We believe that our sample reflects the population of potential end users of MyADHD.

The staged iterative usability tests provided knowledge regarding whether specific tasks could be performed in the intervention and gave direct input on how potential end users would use the intervention. The study was not conducted to identify every single usability problem but rather to show how usability testing with a small sample could identify usability problems that experts did not recognize prior, which allowed us to make significant improvements to the intervention before going full trial. The methods used were effective in identifying elements that needed modification.

### Conclusions

Innovative technologies can play an important role in helping adults with ADHD manage their symptoms better. For such interventions, delivered over the internet without clinician support, to be viable, they need to be developed with the needs, characteristics, and preferences of their intended end users in mind. At the end of a user-centered development process, with usability evaluations, the MyADHD intervention was deemed ready for testing in real-world conditions.

## Appendix

Multimedia Appendix 1 Screenshot of the intervention.

## Abbreviations

ADHD: attention-deficit/hyperactivity disorder
SUS: System Usability Scale
