# Supplementary material for: Internet-Delivered Self-help for Adults With ADHD (MyADHD): Usability Study
Source: JMIR Form Res. 2022 Oct 21;6(10):e37137. doi: 10.2196/37137 (PMC9636529; doi:10.2196/37137)
Supplement: Multimedia Appendix 1 [file formative_v6i10e37137_app1.docx]

Multimedia Appendix. 1

Screenshot of the homepage of the intervention***
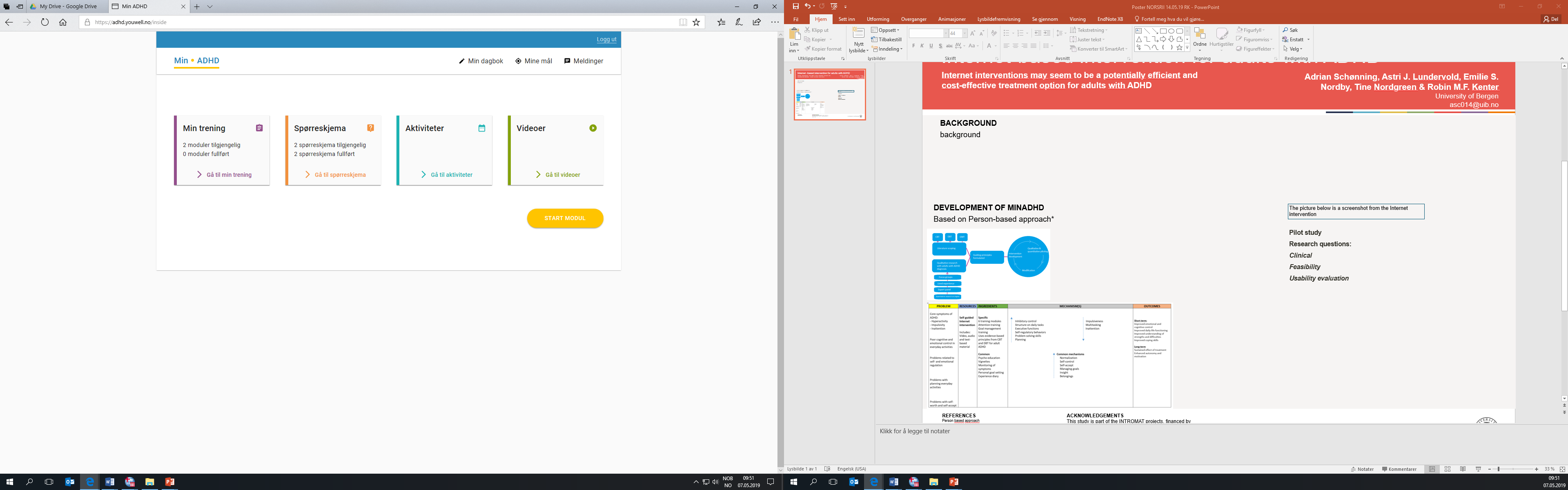
***

**Note** Text is in Norwegian. In the purple box participants find the training modules, iIn the orange box the questionnaires, the blue box the exercises and the green box the videos. In the heading participants could find their dairy, goals and messages.
